# Multiple *Candida* strains causing oral infection in COVID-19 patients under corticosteroids and antibiotic therapy: An observational study

**DOI:** 10.3389/fcimb.2022.1103226

**Published:** 2022-12-23

**Authors:** Farhang Babamahmoodi, Mohammad Sadegh Rezai, Fatemeh Ahangarkani, Ali Mohammadi Kali, Reza Alizadeh-Navaei, Abbas Alishahi, Narges Najafi, Azam Haddadi, Alireza Davoudi, Leila Azargon, Zahra Daftarian, Shirafkan Kordi, Kiana Abbasi

**Affiliations:** ^1^ Antimicrobial Resistance Research Center, Communicable Diseases Institute, Mazandaran University of Medical Sciences, Sari, Iran; ^2^ Pediatric Infectious Diseases Research Center, Communicable Diseases Institute, Mazandaran University of Medical Sciences, Sari, Iran; ^3^ Gastrointestinal Cancer Research Center, Non-communicable Diseases Institute, Mazandaran University of Medical Sciences, Sari, Iran; ^4^ Research Committee, Mazandaran University of Medical Sciences, Sari, Iran; ^5^ Universal Scientific Education and Research Network (USERN), Tehran, Iran; ^6^ Department of Endodontics, Faculty of Dentistry, Dental Research Center, Mazandaran University of Medical Sciences, Sari, Iran; ^7^ Northbay Medical Center, Vacaville Center for Primary Care, Vacaville, CA, United States; ^8^ Department of Microbiology, Zanjan Branch, Islamic Azad University, Zanjan, Iran

**Keywords:** Oral candidiasis, COVID-19, *Candida* species, Corticosteroid, Antibiotic

## Abstract

**Introduction:**

The occurrence of oral candidiasis (OC) is expected in patients with COVID-19, especially those with moderate to severe forms of infection who are hospitalized and may be on long-term use of broad-spectrum antibiotics or prolonged corticosteroid therapy. We aimed to characterize clinical conditions, the prevalence profile of *Candida* species, and outcomes of COVID-19 patients with OC.

**Methods:**

In this observational study, oral samples were obtained from COVID-19 patients suspected of OC admitted to Razi teaching hospital. Patients with OC were monitored daily until discharge from the hospital. Species identification was performed by a two-step multiplex assay named YEAST PLEX, which identifies 17 clinically important uncommon to common yeast strains.

**Results:**

Among the 4133 patients admitted with COVID-19, 120 (2.90%) suffered from OC. The onset of signs and symptoms of OC in patients was, on average (2.92 ± 3.596 days) with a range (of 1-29 days). The most common OC presentation was white or yellow macules on the buccal surface or the tongue. In (39.16%) of patients suffering from OC multiple *Candida* strains (with two or more *Candida* spp.) were identified. The most common *Candida* species were *C. albicans* (60.57%), followed by *C. glabrata* (17.14%), *C. tropicalis* (11.42%), *C. kefyr* (10.83%) and *C. krusei* (3.42%). Notably, OC caused by multiple *Candida* strains was more predominant in patients under corticosteroid therapy (P <0.0001), broad-spectrum antibiotics therapy (P = 0.028), and those who used nasal corticosteroid spray (P <0.0001). The majority of patients who recovered from OC at the time of discharge were patients with OC by single *Candida* species (P = 0.049).

**Discussion:**

Use of corticosteroids and antimicrobial therapy in COVID-19 patients increases risk of OC by multiple *Candida* strains.

## Introduction

Coronavirus Disease 2019 (COVID-19) is an ongoing pandemic with global confirmed cases rising once again. In Iran, a country that started COVID-19 vaccination in February 2021, is now (August 2022) involved in the 7th wave of this infection. Bacterial and fungal co-infections are among the assorted factors that lead to comorbidity and mortality in COVID-19 patients (Gangneux et al., 2020; Macauley and Epelbaum, 2021). Most of these patients have risk factors making them prone to fungal infections, such as hospitalization in the intensive care unit (ICU), high prescription rates of broad-spectrum antibiotics, corticosteroid therapy, use of various catheters, underlying diseases, and immunodeficiencies. Among the opportunistic fungal infections, (e.g., Aspergillosis, Mucormycosis, and Candidiasis) the most common fungal co-infections during previous influenza pandemic outbreaks are now the most common fungal co-infections in COVID-19 patients (Meijer et al., 2020; Salehi et al., 2020; Fortarezza et al., 2021; Kayaaslan et al., 2021; Kayaaslan et al., 2022; Vaseghi et al., 2022).

Due to the undefined standard treatment for COVID-19, side effects of medications, aggressive treatment methods, and combinations of treatment regimens, especially those requiring long-term treatment that suppresses the immune system, some oral disorders such as ulcers, blisters, necrotizing gingivitis, salivary gland alterations, white and erythematous plaques, gustatory dysfunction and oral candidiasis (OC) are widely reported in these patients (Brandini et al., 2021).

Oral candidiasis may be the possible cause of these oral disorders due to excessive colonization of *Candida* species and tissue invasion. As a result of this infection, the patient’s quality of life is also affected due to discomfort and local pain, change in the sense of taste, burning sensation in the mouth, and difficulty breathing and swallowing. It also affects the absorption of liquids and foods consumed by the patients. In addition, OC can progress and involve the esophagus and digestive tract, especially as it can become invasive and spread throughout the bloodstream, causing systemic infections. Timely and accurate diagnosis of OC and accurate identification of its etiological factors in patients suffering from COVID-19 are important to optimize and improve effective treatment (Salehi et al., 2020).

Although *Candida albicans* is the predominant *Candida* species found in patients with OC, there is an increasing incidence of oral colonization and infections caused by non-*albicans Candida* species. Management of candidiasis by non-*albicans Candida* species is challenging due to the resistance pattern of these species to common antifungal agents. Some of these species, including the emergence of multidrug-resistant *C. auris*, are sporadically reported in all continents and cause outbreaks in some cases, presenting a serious global health threat. Also, fluconazole resistance is common in non-albicans *Candida* species such as *C. glabrata*, and *C. krusei*, which are now frequently identified as human pathogens, making treatment of these infections arduous (Laudenbach and Epstein, 2009; Aslani et al., 2018; Saris et al., 2018; Ahangarkani et al., 2020; Arastehfar et al., 2020; Carolus et al., 2021; Chatzimoschou et al., 2021). Moreover, candidiasis caused by mixed *Candida* strains is of great clinical importance, since susceptibility to antifungals differs dramatically among *Candida* species. Limited data on the characterization of OC and the *Candida* species profile in patients with COVID-19 are available. This study characterizes clinical conditions, the prevalence profile of *Candida* species, and outcomes of COVID-19 patients with OC.

## Patients and methods

### Study design and population

This observational cross-sectional study was conducted from March 2021 to March 2022. Census method was performed for sampling. The study involved hospitalized COVID-19 patients over 18 years old who were admitted to Razi teaching hospital (A COVID-19 referral center in Mazandaran province in the north of Iran). The study was approved by the Ethics Committee of the Mazandaran University of Medical Science (IR.MAZUMS.REC.1400. 8977), Sari, Iran. In this study, all applied methods were carried out in accordance with relevant guidelines and regulations. The Census method was performed for sampling. The study population consisted of all confirmed COVID-19 patients with proven OC. The definitive diagnosis of COVID-19 infection was based on the positive results of real-time reverse transcriptase-PCR (RT-PCR) assay for severe acute respiratory syndrome coronavirus-2 (SARS-CoV-2) on nasopharyngeal swabs. Oral candidiasis was confirmed by the direct microscopic identification of *Candida* in the oral samples and isolation in culture. Patients with OC were monitored daily until discharge from the hospital. The following information was collected at enrolment: demographic characteristics, i.e. age and sex; signs and symptoms of COVID-19; medications; the outcome of COVID-19; signs and symptoms of an oral infection; comorbidities, oral hygiene; medications, and outcomes of OC.

### Clinical specimens

In this study, 208 oral samples were aseptically obtained from patients with suspected OC. Specimens were obtained by sterile cotton swabs moistened with normal saline that were placed on the tongue, buccal mucosa, and labial sulcus with rapid rotational movements for ~20 seconds, sealed, and transported in sterile tubes on the same day of collection, to the microbiology laboratory of the hospital and were examined initially in 10% KOH, followed by inoculation on Sabouraud dextrose agar supplemented with 0.5% chloramphenicol, Difco, USA) and CHROMagar *Candida* medium (CHROMagar Company, Paris, France) to ensure purity and incubated at 37°C for 24 h. The plates were examined daily for yeast or yeast-like growth. Plates without mycological growth were discarded after 10 days of incubation and considered negative.

### Fungal identification

Genomic DNA was extracted from 2 to 3-day-old cultures grown on Sabouraud dextrose agar by using an Ultra Clean Microbial DNA Isolation Kit (Mo Bio Laboratories, Carlsbad, CA USA), according to the manufacturer’s instructions, and stored at–20°C prior to use. A two-step multiplex polymerase chain reaction assay named YEAST PLEX that identifies 17 clinically important common to uncommon yeasts (*Candida albicans, Candida dubliniensis, Candida parapsilosis, Candida auris, Candida glabrata, Candida kefyr, Candida krusei, Candida tropicalis, Candida guilliermondii, Candida rugosa, Candida intermedia, Candida lusitaniae, Candida norvegensis, Cryptococcus neoformans, Rhodotorula mucilaginosa, Trichosporon* spp. and *Saccharomyces cerevisiae*) was used according to the instructions as previously described (Aboutalebian et al., 2022). It is notable that the specificity of YEAST PLEX was tested using several reference strains belonging to 17 species and DNA samples of clinically significant non‐target bacteria, parasites, fungi and human genomic DNA. Moreover, the YEAST PLEX method has the ability to identify mixed yeast colonies (Aboutalebian et al., 2022).Sequencing of internal transcribed spacer (ITS) rDNA using primers ITS5 and ITS4 was conducted for strains that weren’t identified by YEAST PLEX multiplex PCR.

### Statistical analysis

Data were analyzed using the SPSS package (version 16.0; Windows, Chicago, IL, USA). The count data are presented as case numbers and percentages. The percentage values in bar graphs were rounded to the nearest whole number. Differences between groups were determined by the Chi-square test or Fisher’s exact test, with P < 0.05 considered to be statistically significant. In cases with a statically significant difference, adjusted odds ratios (OR) with 95% confidence intervals (CIs) were reported.

## Results

**Figure 1:**
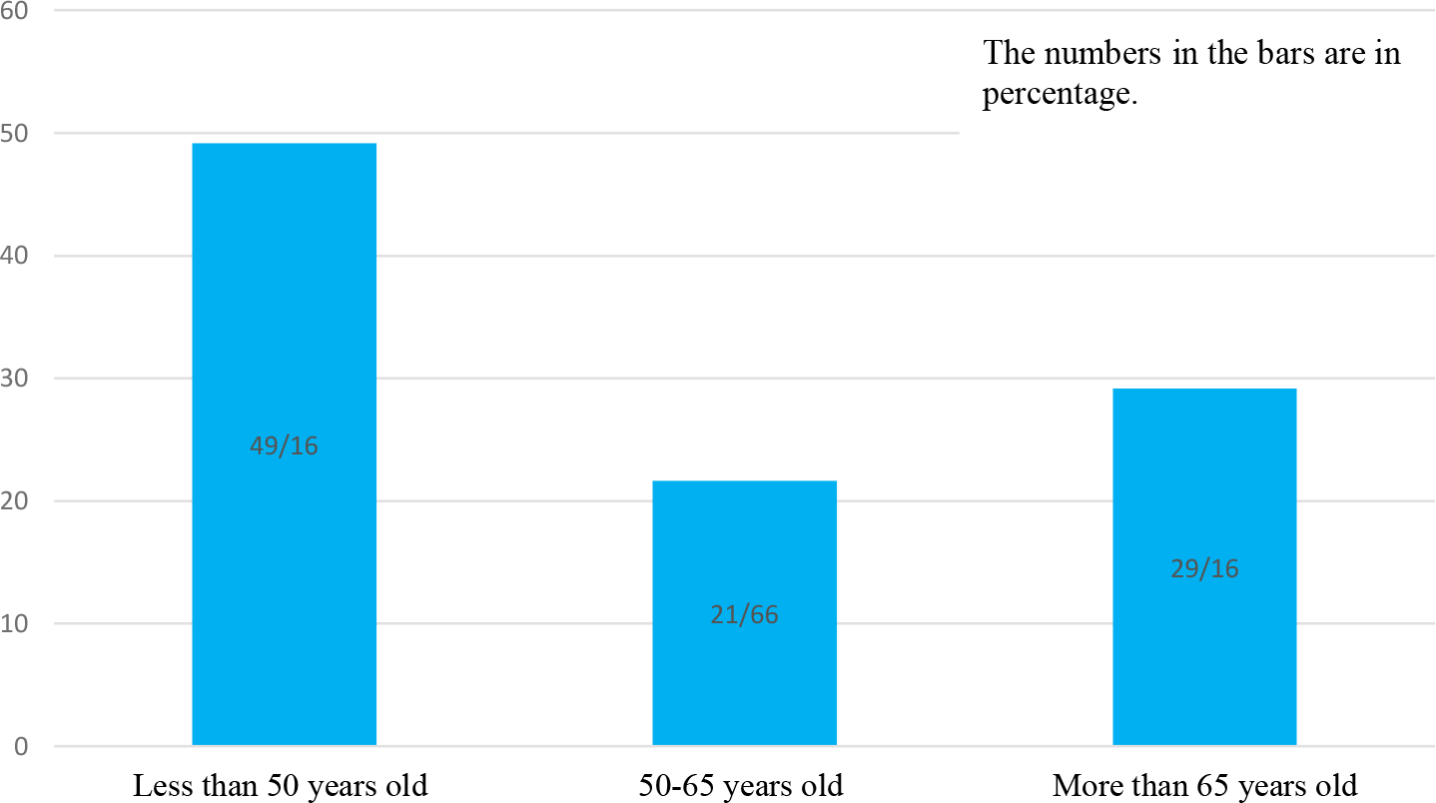
Age Distribution of COVID-19 Patients with Oral Candidiasis.

Among the 4133 patients with COVID-19 admitted during this study, 120 (2.90%) suffered from OC. Demographic characteristics, comorbidities, and clinical features of patients are shown in **Table 1**. Based on age group patients were distributed in three groups including 49.16% (n=59) less 50 years old, 21.66% (n=26) in the range 50-65 years and 29.16% (n=35) more than 65 years old which are illustrated in **Figure 1**.The mean age of the patients was 56.55 ± 15.56 years in the range of (24-96 years old) (n=64; 53.3%) of patients were female and (n=56; 46.7%) were male. The majority of patients had multiple underlying disorders. The most common underlying diseases were diabetes (n=35; 29.2%), hypertension (n=31; 25.8%), and cardiovascular disease (n=26; 21.7%). Also, hyperglycemia during time of admission was seen in (n=17; 14.2%) patients. The most common symptoms were dyspnea (70%), myalgia (65.8%), and fever (55.8%). The most common concomitant medications in patients were Enoxaparin 82.5% and Remdesivir 71.7%. In total, 8.3% of patients were admitted to the ICU, all requiring invasive mechanical ventilation. The average hospitalization stay of patients was 8.22 ± 3.95 days.

**Table 1:** Demographic Features, Comorbidities, Sign and Symptoms, Medication and Outcomes of COVID-19 Patients with Oral Candidiasis.

Demographic	Age (Mean SD)(range) years	56.55 ± 15.56 (24-96)	
		Percentage	Number
	Gender; Male/Female	56/64	46.67/53.3
	Diabetes	35	29.16
	Hypertension	31	25.8
	Cardiovascular disease	26	21.7
	Dyslipidemia	22	18.3
	Hypothyroidism	11	9.2
	Cerebral vascular accident	4	3.3
	Chronic obstructive pulmonary disease	4	3.3
	Chronic kidney disease	3	2.5
	Cancer	3	2.5
	Asthma	2	1.7
	Chronic liver disease	2	1.7
**Sign and symptoms**	Dyspnea	84	70
	Myalgia	79	65.8
	Fever	67	55.8
	No appetite	52	43.3
	Cough	51	42.5
	Chills	36	30
	Productive cough	33	27.5
	Headache	30	25
	Nausea/vomiting	28	23.3
	Diarrhea	18	15
	Chest Pain	17	14.2
	Sore Throat	15	12.5
	Sweating	13	10.8
	C-reactive protein positive	78	65
	Lymphopenia	41	34.2
	Anemia	41	34.2
	Leukopenai	28	23.3
	Thrombocytopenia	12	10
	Neutropenia	2	1.7
	Nsaids	120	100
**Medications for COVID-19**	All Corticosteroid therapy	107	89.16
	IV corticosteroid therapy	104	86.66
	Enoxaparin	99	82.5
	Antiviral therapy	86	71.7
	Broad spectrum antibiotics	66	55
	Spray corticosteroid therapy	36	30
**Outcomes**	ICU admission	10	8.3
	Invasive mechanical ventilation	10	8.3
	Non-invasive mechanical ventilation	105	87.5
	Duration of hospitalization (days): Mean ± SD	8.22 ± 3.95	
	All-cause mortality	1	0.8

Oral candidiasis clinical manifestations, comorbidities, oral hygiene, and healthcare-associated factors for higher risk of OC in patients are illustrated in **Table 2**. The onset of signs and symptoms of OC in patients was on average (2.92 ± 3.596 days) with a range of 1-29 days.

**Table 2:** Oral candidiasis clinical manifestations, comorbidities, social state, and healthcare-associated factors of patients with oral candidiasis caused by single or multiple *Candida* strains.

Variables	Total	Single *Candida* strains N (%)	Multiple *Candida* strains N (%)	*P value*
Demographic				
Age (>65 years)	35	21 (28.76)	14 (29.78)	0.904
Gender; Male/Female	56/64	36/37 (49.31/50.68)	20/27 (46.51/62.98)	0.469
The onset of signs and symptoms (day)	4.92 ± 3.596	5.25 ± 4.235	4.4 ± 2.223	0.212
Oral Candidiasis Presentation				
White or yellow macule on the buccal surface	79	47 (64.38)	32 (68.08)	0.676
White or yellow macule on the tongue	62	39 (53.42)	23 (48.93)	0.631
Xerostomia	39	25 (34.24)	14 (29.78)	0.611
White or yellow macule on the soft palate	33	18 (24.65)	15 (31.91)	0.385
Irritation	26	15 (20.54)	11 (23.4)	0.711
Atrophy of tongue	14	7 (9.58)	7 (14.89)	0.377
Erythematous patch on the tongue	10	5 (6.84)	5 (10.63)	0.464
White or yellow macule on the gums	8	6 (8.21)	2 (4.25)	0.395
White or yellow macule on the lips	4	1 (1.36)	3 (6.38)	0.135
Perioral fissures	1	1 (1.36)	0 (0)	0.420
White or yellow macule on the pharynx	1	0 (0)	1 (2.12)	0.211
Oral Hygiene and Social Status				
No mouth washing	100	61 (83.56)	39 (82.97)	0.933
No teeth brushing	77	48 (65.75)	29 (61.7)	0.651
Decayed teeth	57	40 (54.79)	17 (36.17)	**0.046**
Missing teeth	52	36 (49.31)	16 (34.04)	0.099
Dentures	40	19 (26.02)	21 (44.68)	**0.034**
Smoking	16	10 (13.69)	6 (12.76)	0.883
Underlying Diseases				
Diabetes mellitus	35	22 (30.13)	13 (27.65)	0.771
hypertension	31	18 (24.65)	13 (27.65)	0.714
Cardiovascular disease	26	14 (19.17)	12 (25.53)	0.410
Healthcare Associated Factors				
Corticosteroid therapy duration (day)	4.44 ± 4.82	1.137 ± 0.732	7.978 ± 5.289	**<0.0001**
Corticosteroid therapy	107	60 (82.19)	47 (100)	**0.028**
IV Corticosteroid therapy	104	57 (78.08)	47 (100)	**<0.0001**
Antiviral therapy	86	54 (73.97)	32 (68.08)	0.537
Nasal tube oxygen therapy	74	47 (64.38)	27 (57.44)	0.446
Mask oxygen therapy	70	46 (63.01)	24 (51.06)	0.195
Broad spectrum antibiotics	66	23 (31.08)	43 (93.47))	**<0.0001**
Spray Corticosteroid therapy	36	7 (9.6)	29 (61.7)	**<0.0001**
ICU admission	10	5 (6.84)	5 (10.63)	0.464
Mechanical ventilation	10	5 (6.84)	5 (10.63)	0.464
Treatment and Outcome of Oral Candidiasis				
Nystatin Suspension	102	66 (90.4)	36 (76.6)	**0.039**
Mouthwash contain nystatin	14	7 (9.58)	7 (14.89)	0.396
Fluconazole Infusion	2	0 (0)	2 (4.25)	1.000
No treatment	2	2 (2.72)	0 (0)	0.268
Cured at discharge time	103	59 (80.82)	20 (42.6)	**<0.0001**
Not cured at discharge time	17	14 (18.91)	27 (57.4)	

Significance is shown in boldface.

White or yellow macules were present on the buccal surface (n=79; 65%), the tongue (n=62; 51.66%), soft palate (n=33; 27.5%), gums (n=8; 6.66%), lips (n=4; 3.33%) and on the pharynx (n=1; 0.83%). Other presentations included, xerostomia (n=39; 32.5%), irritation (n=26; 21.66%), atrophy of the tongue (n=14; 11.66%), erythematous patches on the tongue (n=10; 8.33%) and perioral fissures (n=1; 0.83%). A majority of patients did not observe oral hygiene (n=100; 83.33% mouthwash, n=77; 64.16% teeth brushing) before OC presentation. Also n=57; 47.5% patients had at least one decayed and tooth (n=40; 33.33%) had dentures. Moreover, n=16; 13.33% of patients were cigarette smokers. Healthcare-associated factors for higher risk of OC were: corticosteroids (n=102; 85%), nasal tube oxygen therapy (n=74; 61.66%), oxygen mask (n=70; 58.33%), broad-spectrum antibiotics (n=66; 55%) and corticosteroid spray use (n=36; 30%). Nystatin suspension (n=102; 85%), mouthwash containing nystatin (n=14; 11.66%), and fluconazole (n=2; 1.66%) were administrated for patients, while (n=2; 1.66%) didn’t receive antifungal therapy. Oral candidiasis was cured in (n=103; 85.83%) of patients at discharge, while (n=17; 14.16%) of patients were not cured at time of discharge.

**Figure 2:**
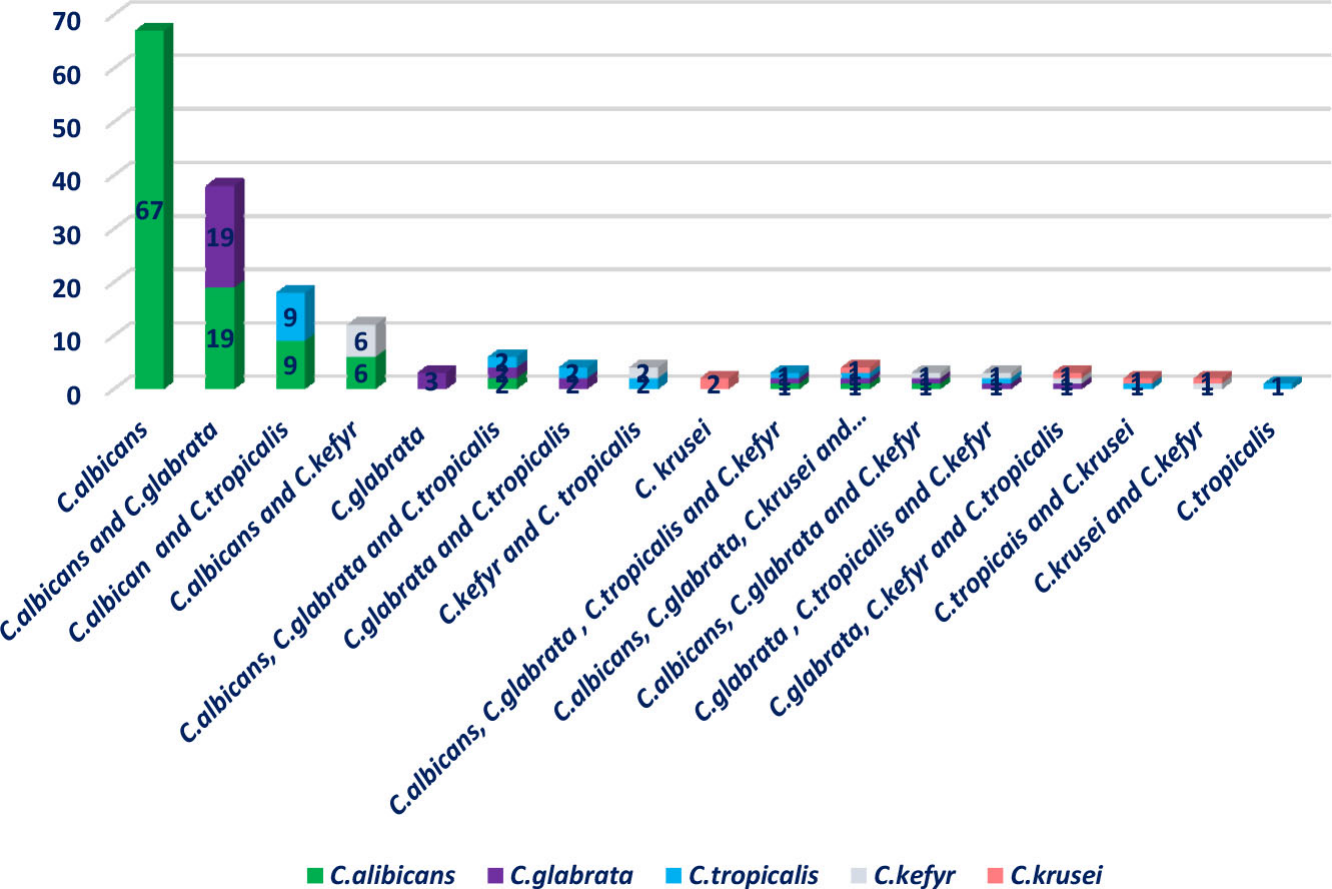
Distribution of *Candida* species causing oral candidiasis based on single or multi-*Candida* species.

In total, n=47; 39.16% of patients suffered from OC caused by multiple *Candida* strains (with 2 or more *Candida* spp.). Distribution of *Candida* species causing OC based on single or multiple *Candida* strains is illustrated in **Figure 2**.

In this study, 175 strains of *Candida* species isolated from 120 patients were diagnosed as causative agents of oral candidiasis. The most common *Candida* species were *C. albicans* (n = 106; 60.57%), followed by *C. glabrata* (n = 30; 17.14%), *C. tropicalis* (n = 20; 11.42%), *C. kefyr* (n = 13; 10.83%) and *C. krusei* (n = 6; 3.42%). Oral candidiasis clinical manifestations, comorbidities, and healthcare-associated factors were not significantly different between patients infected by single *Candida* species vs multiple *Candida* strains. Oral candidiasis caused by single *Candida* strains was significantly higher in patients with decayed teeth (OR 2.17, 95% CI 1–4.707, P = 0.046). In addition, OC caused by multiple *Candida* strains was considerably more in patients with dentures (OR 1.171, 95% CI 0.986–1.391, P = 0.034). Significantly, OC caused by multiple *Candida* strains was more predominant in patients under corticosteroid therapy (OR 4.185, 95% CI 1.047–17.714, P <0.0001), broad-spectrum antibiotics (OR 4.078, 95% CI 2.238–7.429, P = 0.028), and those using inhaled corticosteroid sprays (OR 2.361, 95% CI 1.63–3.419, P <0.0001). Notably, the majority of patients which recovered from OC at the time of discharge from the hospital were patients with candidiasis by single *Candida* species (OR 3.005, 95% CI 0.912–9.895, P = 0.049). Also, the frequency of *Candida* species causing OC in patients undergoing corticosteroid and antibiotic therapy versus non-users is shown in **Table 3**.

**Table 3:** Frequency of *Candida* species causing oral candidiasis in Patients Undergoing Treatment with Systemic corticosteroids and antibiotics versus Non-Users.

Species	Corticosteroid Users (n=107)		P value	Antibiotics users(n=66)		P value
	Yes	No	0.014	Yes	No	<0.0001
*C. albicans*	55 (51.40)	12 (11.21)		17 (25.75)	50	
*C.glabrata*	3 (2.80)	0		3 (4.54)	0	
*C.krusei*	2 (1.86)	0		2 (3.03)	0	
*C.tropicalis*	1 (0.09)	0		1 (1.51)	0	
Multi *Candida* species	46 (42.99)	1		43 (65.15)	4	

Significance is shown in boldface.

## Discussion

Oral candidiasis is unusual in healthy adults. People at higher risk for getting OC include neonates and individuals with at least one of the risk factors, such as wearing dentures, diabetes, cancer, HIV/AIDS, taking antibiotics or corticosteroids, taking medications that cause dry mouth, and smoking. The occurrence of OC should be anticipated in patients with COVID-19, especially those with moderate to severe forms of infection who are hospitalized and may be on long-term use of broad-spectrum antibiotics or prolonged corticosteroid therapy.

In the current study, during one year, 2.9% of 4133 patients admitted to the COVID-19 referral center in the north of Iran suffered from OC at time of admission. Consistent with other studies, *C. albicans* was the predominant, isolated species in these patients (Aslani et al., 2018; Salehi et al., 2020). However, it is interesting that 39.16% of these patients suffered from OC caused by two or more *Candida* spp. The compromising host immune status that affects the natural defenses that suppress the growth of invading pathogens results in multiple *Candida* species infections. Also, it has been seen in immunocompromised patients that the frequency of infections involving mixed fungal genera would be similar to that of disorders involving mixed fungal species due to severe immunosuppression, which accelerates colonization of *Candida* strains and boosts the ability of independent pathogens to penetrate tissue Soll, 2022.

In this study, 89% of patients were under corticosteroid therapy, and 55% used broad-spectrum antibiotics. It was found that corticosteroid therapy and use of broad-spectrum antibiotics were significantly higher in patients with OC caused by two or more *Candida* spp. In addition, the duration of corticosteroid therapy was greater in these patients compared to patients with OC caused by one *Candida* species. Similar to our findings, Xia et al. reported corticosteroid therapy caused an increased prevalence of OC by non-albicans strains (Xiao et al., 2020). Moreover, Nambiar et al. noted that the development of OC in COVID-19 patients could be due to prolonged mechanical ventilation in the ICU and the long-term use of broad-spectrum antibiotics. (Nambiar et al., 2021). It is noted that the possibility of a potential of OC caused by empirical broad‐spectrum antibiotics prescription in a mild or moderate form of COVID‐19 case should also be considered (Riad et al., 2022). Ahmed et al., in a review article, reported a direct correlation between the development of candidiasis with the use of antibiotics and corticosteroids in COVID-19 patients (Ahmed et al., 2022).

The profile of *Candida* species causing OC in our patients did not changed during the COVID-19 pandemic, and albeit of antibiotics overuse and corticosteroids in patients, emerging uncommon *Candida* species was not observed in our study (Aslani et al., 2018; Shokohi et al., 2018; Arastehfar et al., 2020). *Candida glabrata*, *C. tropicalis*, *C. kefyr* and *C. krusei* were the most prominent non-albicans *Candida* species isolated from patients with OC caused by multiple *Candida* species. Notably, these non-*albicans Candida* species are intrinsically azole resistant or low susceptibility to azole antifungals (Shokohi et al., 2018; Ahangarkani et al., 2019; Ahangarkani et al., 2020). Colonization of oral mucosa with *C. glabrata* is common in cancer patients (Aslani et al., 2018). The prevalence of *C. glabrata* in our study was higher compared to the Khalil et al. study in Egypt and Salehi’s study in Iran. However, *C. tropicalis* prevalence was consistent with the study by Khalil et al., and the prevalence of *C. krusei* was similar to the findings of Salehi’s study (Salehi et al., 2020; Khalil et al., 2022).

As OC is an indirect indicator of cell-mediated immunodeficiency and has a high predictive value for invasive candidiasis in immunocompromised patients, and the lack of fungal identification methods to species level in low-income countries such as Iran, invasive candidiasis caused by these species in patients exposed to high-risk medications such as patients with moderate to severe form of COVID-19 is noteworthy. The current study has some limitations. Since this observational study was performed on COVID-19 patients with OC, ideally, to obtain insights into the epidemiological status, it was better to compare COVID-19 patients with OC with a control group, such as COVID-19 patients without any co-fungal infection. Moreover, the occurrence of OC in patients with a mild and moderate form of COVID-19 should be investigated. Furthermore, several OC caused by multiple *Candida* species were observed, antifungal susceptibility testing should be performed for all isolates deemed clinically significant. Also, this study endorses the involvement of dental practitioners among the treatment teams dealing with COVID‐19 patients.

## Conclusion

Although the profile of *Candida* species causing OC in our patients did not changed during the COVID-19 pandemic, overconsumption of corticosteroids and antimicrobial therapy in COVID-19 patients could result in OC by multiple *Candida* strains.

## Data availability statement

The original contributions presented in the study are included in the article/Supplementary Materials, further inquiries can be directed to the corresponding author/s.

## Ethics statement

The study was approved by the Ethics Committee of the Mazandaran University of Medical Science (IR.MAZUMS.REC.1400. 8977), Sari, Iran and was performed in compliance with the Declaration of Helsinki. The patients/participants provided their written informed consent to participate in this study.

## Author contributions

FB, MR, FA and AK designed the project, collected data, wrote and performed the critical review of the manuscript. FB, MR, AK, RA-N, NN, AA, AH, LA, SK, KA, AD, ZD and FA contributed to clinical data collection. AA and FA carried out statistical interpretation. All authors contributed to the article and approved the submitted version.
